# Visible – Near infrared hyperspectral dataset of healthy and infected apple tree leaves images for the monitoring of apple fire blight

**DOI:** 10.1016/j.dib.2023.109532

**Published:** 2023-08-29

**Authors:** Belal Gaci, Florent Abdelghafour, Maxime Ryckewaert, Silvia Mas-Garcia, Marine Louargant, Florence Verpont, Yohana Laloum, Aude Moronvalle, Ryad Bendoula, Jean-Michel Roger

**Affiliations:** a CTIFL, France; b ITAP-INRAE, Institute Agro, University Montpellier, Montpellier, France; c ChemHouse Research Group, Montpellier, France

**Keywords:** Hyperspectral images, Disease detection, Fire blight on apple trees, Epidemiological monitoring

## Abstract

This dataset consists of three groups of hyperspectral images of apple tree plants. The first group of images consists of a temporal monitoring of seven apple tree plants, infected with fire blight (*Erwinia amylovora)*, and six control plants over a period of 15 days. The second group of images includes a temporal monitoring of three infected plants, seven plants subjected to water stress, and seven control plants. The third group of images corresponds to acquisitions made in the orchard on nine trees showing symptoms of fire blight and six control trees. The pixel locations of infected areas have been provided for all images featuring symptomatic plants.

**Specifications Table**

**Table:** 

Subject	Hyperspectral imaging; Multivariate analysis; Pathology. Agronomy;
Specific subject area	Plant Sciences; Disease detection
Data format	RAW
Type of data	VIS-NIR Hyperspectral Images, RGB images, Annotation Files (csv format)
Data collection	The hyperspectral images were acquired using a hyperspectral camera (Specim IQ, Specim Imaging LTD, Finland) with a spectral range from 400 nm to 1000 nm, a spectral resolution of 3 nm, and a spatial resolution of 512 × 512 pixels.
Data source location	Controlled environment acquisition, under greenhouse: CTIFL of Lanxade, Prigonrieux, France Orchard acquisition: Plan-d'Orgon, France
Data accessibility	Repository name: Visible - Near infrared hyperspectral dataset of healthy and infected apple tree leaves images for the monitoring of apple fire blight Data identification number: https://doi.org/10.57745/R6AMN3 Direct URL to data: Visible - Near infrared hyperspectral dataset of healthy and infected apple tree leaves images for the monitoring of apple fire blight. - Data INRAE

## 1 Value of the Data

• The data presents a temporal monitoring of apple tree plants, infected or not with fire blight, in a sequence of hyperspectral images. Therefore, it enables other researchers to study the potential of hyperspectral imaging for early disease detection [1,2].
• This dataset is intended for researchers and developers of digital solutions for agriculture to test new methods (variable selection, data exploration, regression methods) or to have reference data to guide their future experiments.
• This dataset can be used to build disease classification models, distinguishing between symptomatic and non-symptomatic classes, as well as the class subjected to water stress.

## 2 Data Description

The data is organized into three groups:

• Data acquired in March 2021: the images were acquired under laboratory conditions with 7 plants inoculated with fire blight on leaves and 6 control plants.
• Data acquired in July 2021: the images were acquired under laboratory conditions with 3 plants inoculated with fire blight on leaves, 7 plants subjected to water stress, and 7 control plants.
• Data acquired in May 2022: the images were acquired in the orchard and contain 9 plants showing symptoms of fire blight and 6 control plants without symptoms.

**Fig. 1:**
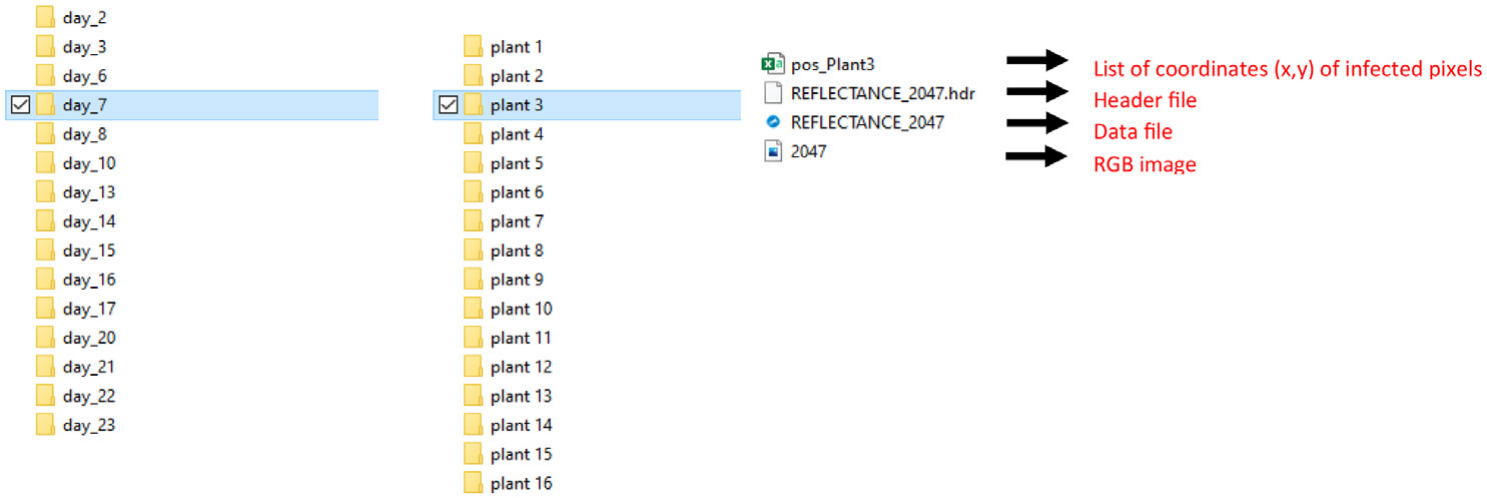
Schéma illustrant l'organisation du stockage des données.

The data is organized into folders that include the month and year of acquisition. For the images acquired under laboratory conditions, each folder contains subfolders named according to the day of acquisition relative to the first day of inoculation. Inside each subfolder, there are folders labelled with the plant number, each containing four files (see Fig. 1):

- An “.HDR” header file containing metadata describing the image format and enabling proper data reading.
- A “.DAT” file containing the binary data of the hyperspectral image.
- A “.PNG” file providing an RGB view of the acquired image.
- A “.CSV” file containing the positions of symptomatic pixels for the inoculated plants. This file has two columns: the first indicates the vertical position of the pixel, while the second indicates the horizontal position of the pixel.

For the images acquired in the orchard, inside the corresponding folder, there are subfolders labelled with the plant number, also containing the same four files: a “.HDR” file, a “.DAT” file, a “.PNG” file, and a “.CSV” file with the positions of symptomatic pixels.

### 2.1 Data Acquired in March 2021

The laboratory data from March 2021 consists of a temporal series of hyperspectral images of apple tree plants inoculated on the leaves, as well as control plants. These images are divided into 3 subsets:

• The first subset includes images of inoculated plants 1, 2, 3, 4, as well as images of control plants 8 and 9. These images were acquired from day 4 to day 15 after inoculation, with missing images from day 9 to day 11. The first signs of fire blight are visible to the naked eye as early as the 5th day on these plants.
• The second subset contains images of inoculated plants 5, 6, and 7, as well as images of control plants 10 and 11. The images were acquired from day 1 to day 15 after inoculation, with the first visible signs to the naked eye appearing on the 7th day for these plants.
• The third subset includes images of control plants 12 and 13. The images were acquired from day 1 to day 15 after inoculation, with missing images for days 3, 12, and 13.

The positions of the fire blight spots were extracted for each infected leaf of the inoculated plants, from the appearance of the symptoms. The Table 1 summarizes the acquisitions of the different plants based on the number of days after inoculation.

**Table 1:** The acquisitions of the different plants based on the number of days after inoculation for the data acquired in March 2021.

Plants		Days														
		1	2	3	4	5	6	7	8	9	10	11	12	13	14	15
Inoculated plants or plants with symptoms																
SS 1	Plant 1				x	x	x	x	x				x	x	x	x
	Plant 2				x	x	x	x	x				x	x	x	x
	Plant 3				x	x	x	x	x				x	x	x	x
	Plant 4				x	x	x	x	x				x	x	x	x
SS 2	Plant 5	x	x	x	x	x	x	x	x	x	x	x	x	x	x	x
	Plant 6	x	x	x	x	x	x	x	x	x	x	x	x	x	x	x
	Plant 7	x	x	x	x	x	x	x	x	x	x	x	x	x	x	x
Control plants																
SS 1	Plant 8				x	x	x	x	x				x	x	x	x
	Plant 9				x	x	x	x	x				x	x	x	x
SS 2	Plant 10	x	x	x	x	x	x	x	x	x	x	x	x	x	x	x
	Plant 11	x	x	x	x	x	x	x	x	x	x	x	x	x	x	x
SS 3	Plant 12	x	x		x	x	x	x	x	x	x	x			x	x
	Plant 13	x	x		x	x	x	x	x	x	x	x			x	x

It should be noted that, although some plants showed symptoms, it was difficult to locate the symptomatic pixels on the inoculated leaves. Consequently, the position of these pixels is not provided.

Table 2 summarizes the acquisitions of the different plants based on the number of days after inoculation.

**Table 2:** The acquisitions of the different plants based on the number of days after inoculation for the data acquired in March 2021.

Plants	Days														
	2	3	6	7	8	10	13	14	15	16	17	20	21	22	23
Inoculated plants or plants with symptoms															
Plant 1	x	x	x	x	x	x	x	x	x	x	x	x	x	x	x
Plant 2.1	x	x	x	x	x	x	x	x	x	x	x	x	x	x	x
Plant 2.2												x	x	x	x
Plant 2.3												x	x	x	x
Plant 2.4												x	x	x	x
Plant 2.5												x	x	x	x
Plant 3	x	x	x	x	x	x	x								
Control plants															
Plant 4	x	x	x	x	x	x	x	x	x	x	x	x	x	x	
Plant 5	x	x	x	x	x	x	x	x	x	x	x	x	x	x	x
Plant 6	x		x	x	x	x	x	x	x	x	x	x	x	x	x
Plant 7	x	x	x		x	x	x	x	x	x	x	x	x	x	x
Plant 8	x	x	x	x		x	x	x	x	x	x	x	x	x	x
Plant 9	x	x	x	x	x	x	x	x	x	x	x				
Plant 10	x	x	x	x	x	x	x	x	x	x	x	x	x	x	x
Plants under water stress															
Plant 11	x	x	x	x	x	x	x	x	x	x	x	x	x	x	x
Plant 12	x		x	x	x	x	x	x	x	x	x	x	x	x	x
Plant 13	x	x	x	x	x		x	x	x	x	x	x	x	x	x
Plant 14	x	x	x	x	x	x	x	x	x	x	x	x	x	x	x
Plant 15	x	x	x	x	x	x	x	x	x	x	x	x	x	x	
Plant 16	x	x	x	x	x	x	x	x	x	x	x	x	x	x	x
Plant 17	x	x	x	x	x	x	x	x	x	x	x	x	x	x	x

**Fig. 2:**
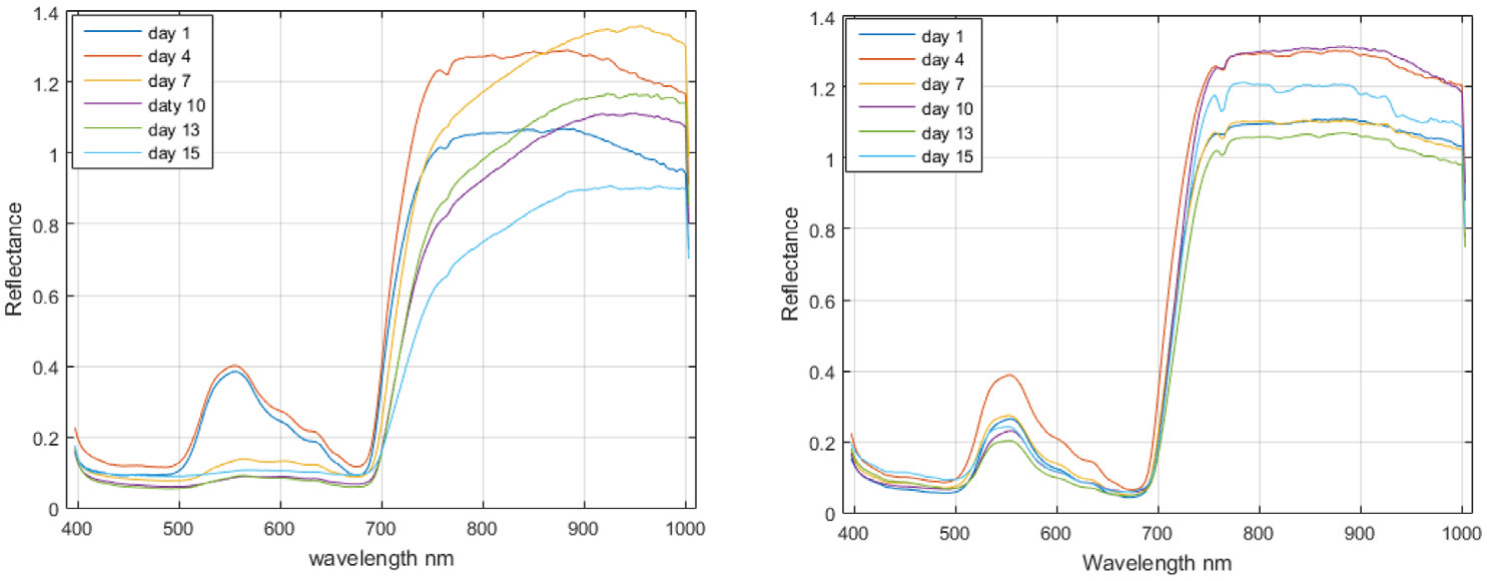
Average spectra of inoculated leaf plant 6 (left) and non-inoculated plant 11 (right) for the dates 1, 4, 7, 10, 13, and 15 days after inoculation.

Fig. 2 illustrates the temporal evolution of the average spectra for plant 6, which was inoculated on the leaf, and plant 11, which was not inoculated. The spectra were recorded on different acquisition dates. It is noticeable that the shape of the average spectra for the inoculated plant varies considerably with the acquisition date, in contrast to the relatively stable average spectra of the non-inoculated plant. This discrepancy is particularly pronounced in the region encompassing the diminished chlorophyll peak and the plateau between 750 nm and 900 nm, where a distinct transition from a plateau to a slope can be observed.

### 2.2 Data Acquired in July 2022

The laboratory data from July 2022 presents a temporal monitoring over a period of 23 days for apple tree plants inoculated on leaves, plants subjected to water stress, and control plants. The images were acquired from day 2 to day 23 after inoculation, encompassing 3 leaf-inoculated plants, 6 control plants, and 8 plants subjected to water stress. The acquisitions were done on weekdays, which explains the absence of images for weekends. Fire blight symptoms are visible to the naked eye as early as the 7th day following inoculation, and the positions of spots were extracted for each infected leaf of the 3 inoculated plants, starting from the appearance of symptoms.

It should be noted that the inoculated leaf 2.1 of plant 2 was monitored for 23 days, while 4 other symptomatic leaves of the same plant (2.2, 2.3, 2.4, 2.5) were monitored from day 20 to day 23 after inoculation. The table below summarizes the observations made based on the number of days after inoculation.

Furthermore, it should be mentioned that the inoculated leaf of plant 3 fell on the 14^th^ day, which explains the absence of acquisitions from that date.

**Fig. 3:**
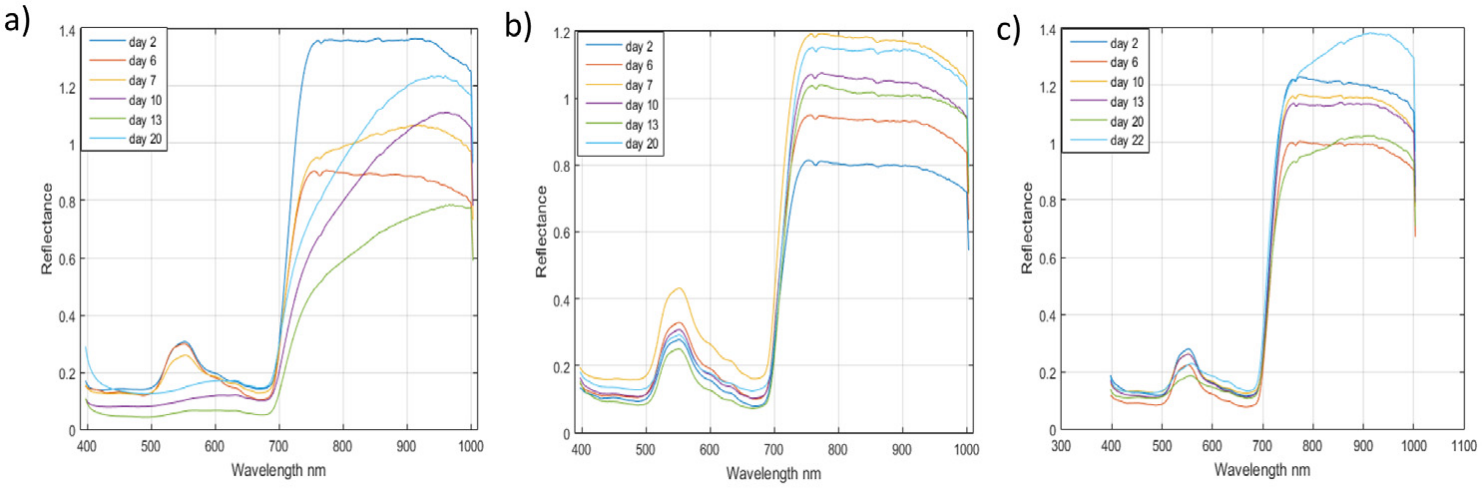
Mean spectrum of inoculated plant 2 (Fig. A) and healthy plant 4 (Fig. B) at different dates after inoculation (2, 6, 7, 10, 13, and 20), and the mean spectrum of plant 11 (Fig. C) under water stress at dates (2, 6, 10, 13, 20 and 22).

**Fig. 4:**
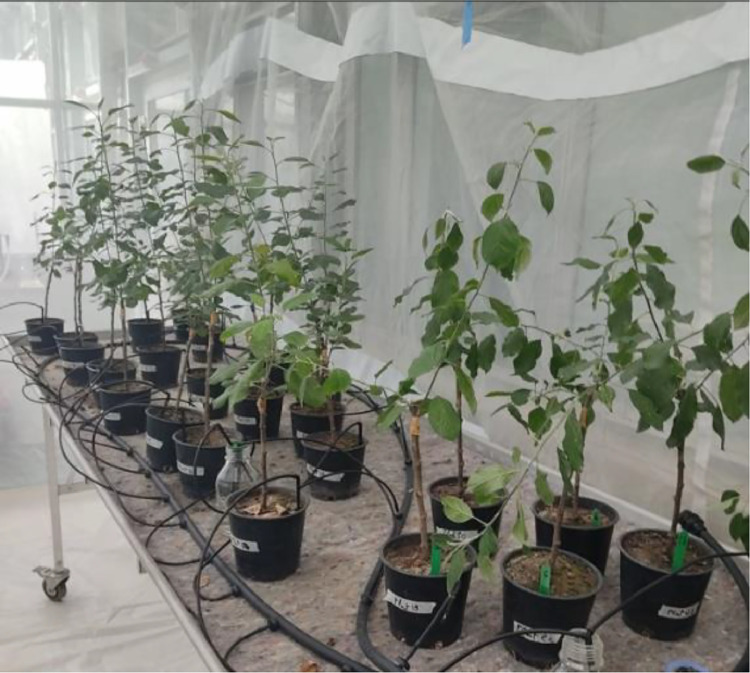
Bench-grafted Chantecler variety plants artificially inoculated in a confined greenhouse.

For example, Fig. 3 (a), (b), and (c) respectively show the evolution of the average spectra for leaf-inoculated plant 2, non-inoculated plant 4, and plant 11 under water stress, based on the acquisition date. It is possible to observe that the shape of the average spectra for plant 2 and plant 11 varies differently depending on the acquisition date, mainly in the spectral ranges between 500 and 680 nm, as well as between 700 and 900 nm. On the other hand, the shape of the average spectrum for non-inoculated plant 4 remains stable regardless of the acquisition date. Fig. 4 shows the acquisition conditions in the laboratory.

### 2.3 Data Acquired in the Orchard

The data acquired in the orchard in May 2022 involves apple tree plants exhibiting symptoms of bacterial fire blight on leaves, as well as non-symptomatic plants. The images were acquired over the course of a single day from 9 am to 4 pm, encompassing 9 plants with symptomatic leaves and 6 non-symptomatic plants. The positions of symptomatic pixels were extracted for each infected leaf of the 8 symptomatic plants.

## 3 Experimental Design, Materials and Methods

### 3.1 March 2021 Acquisitions

In order to monitor the progression of fire blight symptoms, artificial inoculations were conducted on bench-grafted apple plants in a level 2 confined greenhouse module at CTIFL in Lanxade, Prigonrieux, France. Three trials were conducted on 13 bench-grafted apple plants of the "Chantecler" variety. The *Erwinia amylovora* strain CFBP3472 was inoculated at a concentration of 2 × 10^^12^ cfu/ml by incising the leaves with a knife [3]. The three biological trials were conducted successively with a one-week interval. The first trial consisted of 6 plants, including 4 plants inoculated on leaves and 2 non-inoculated plants as controls. The second biological trial consisted of 5 plants, including 3 plants inoculated on leaves and 2 non-inoculated plants as controls. The third trial consisted of 2 non-inoculated plants as controls.

### 3.2 July 2022 Acquisitions

In order to monitor the progression of fire blight symptoms, artificial inoculations were conducted on potted grafts in a level 2 confined greenhouse module at CTIFL in Lanxade, Prigonrieux, France. Three trials were performed on 17 potted Gala apple tree plants. The strain CFBP3472 of *Erwinia amylovora* was inoculated at a concentration of 106 cfu/ml by making incisions on the leaves using a knife. This trial consisted of 17 plants, including 3 plants inoculated on the leaves, 6 non-inoculated plants as controls, and 8 plants subjected to water stress.

### 3.3 Orchard Acquisition in May 2022

The image acquisitions were carried out in Plan d'Organ, France, in an orchard of "Rosy Glow" apple trees. The acquisitions were done in a single day on the leaves of 9 plants showing symptoms of fire blight and 6 symptom-free plants.

### 3.4 Image Acquisition Method

**Fig. 5:**
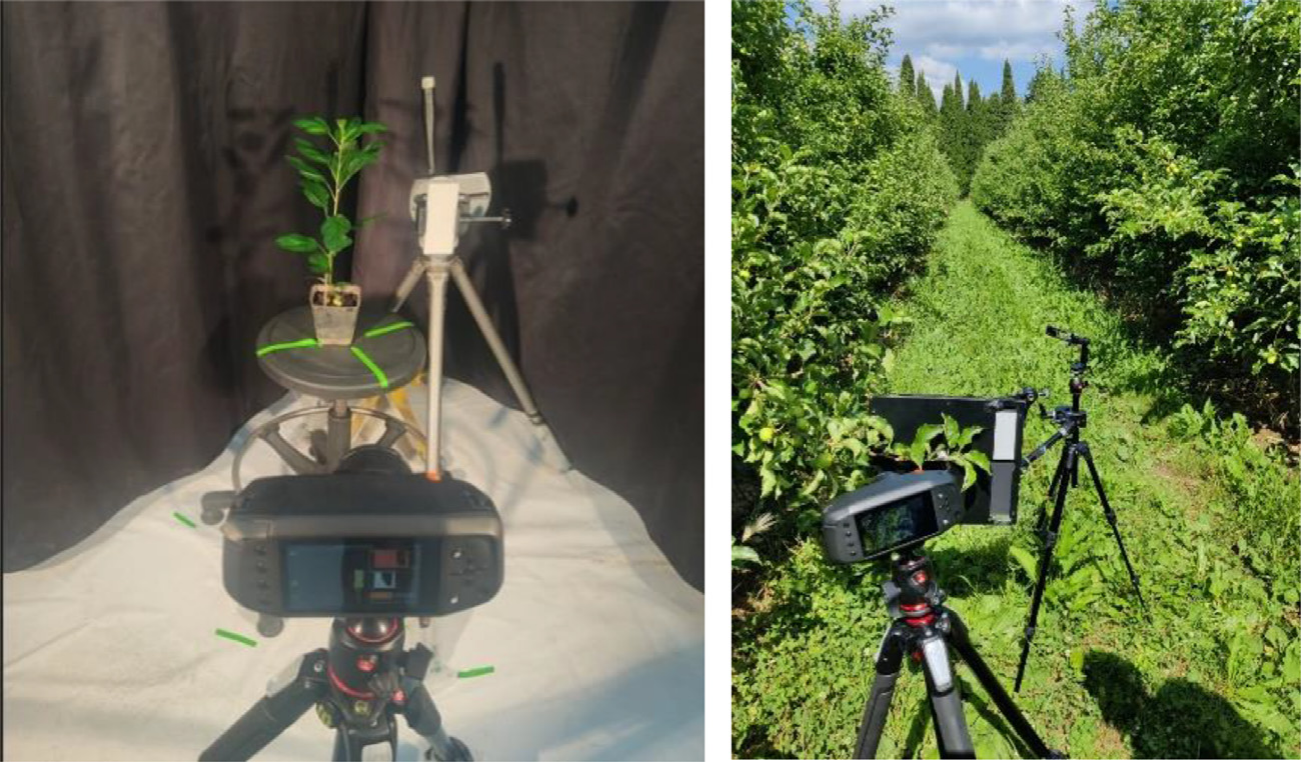
Hyperspectral image acquisition setup. On the left, in laboratory conditions, and on the right, in the orchard.

The different image acquisition campaigns were conducted using a SPECIM IQ camera (Specim spectral imaging LTD. ©) (Fig. 5), which offers a resolution of 512*512 pixels and 204 wavelengths covering a spectral range from 397 nm to 966 nm. A reference (SRS50, Spectralon®) was placed near the scene to correct for variations in brightness during image processing. For the March 2021 campaign, the acquisition distance was 50 cm, and a black curtain was placed behind the plant to avoid potential interactions with the background. Two halogen lamps (Ref ampoule: Single Ended Halogen - HPL HPL 575W 240V 88477) were positioned one meter away from the plant at an incident angle of 45° to provide uniform illumination of the scene. The same conditions were used for the July 2022 campaign, except for the camera-to-plant distance, which was 25 cm, and the replacement of the black curtain with a black panel. For the May 2022 acquisitions in the orchard, the same conditions as the laboratory acquisitions in July 2022 were employed, except for the lighting, which was natural. All images were automatically corrected for reflectance by the camera.

### 3.5 Limitations

None.

## Ethics Statement

The authors confirm that they have adhered to the ethical requirements for publication in Data in Brief.

The authors confirm that their research did not involve human subjects.

The authors confirm that no animal experiments were carried out as part of this work.

The authors confirm that their research did not utilize data sourced from social media platforms.

## CRediT authorship contribution statement

**Belal Gaci:** Conceptualization, Methodology, Software, Writing – review & editing. **Florent Abdelghafour:** Writing – review & editing, Supervision. **Maxime Ryckewaert:** Writing – review & editing. **Silvia Mas-Garcia:** Writing – review & editing. **Marine Louargant:** Supervision, Writing – review & editing. **Florence Verpont:** Supervision. **Yohana Laloum:** Methodology, Resources, Supervision. **Aude Moronvalle:** Resources. **Ryad Bendoula:** Supervision. **Jean-Michel Roger:** Supervision.

## Data Availability

Visible - Near infrared hyperspectral dataset of healthy and infected apple tree leaves images for the monitoring of apple fire blight. (Original data) (data.gouv).
